# Personality and Intelligence Interact in the Prediction of Academic Achievement

**DOI:** 10.3390/jintelligence6020027

**Published:** 2018-05-10

**Authors:** Sebastian Bergold, Ricarda Steinmayr

**Affiliations:** Department of Psychology, Technical University Dortmund, Emil-Figge-Straße 50, 44227 Dortmund, Germany; ricarda.steinmayr@tu-dortmund.de

**Keywords:** personality factors, personality facets, Big Five, intelligence, personality-intelligence interface, academic achievement, grade point average

## Abstract

Personality predicts academic achievement above and beyond intelligence. However, studies investigating the possible interaction effects between personality and intelligence when predicting academic achievement are scarce, as is the separate investigation of broad personality factors versus narrow personality facets in this context. Two studies with 11th grade students (Study 1: *N* = 421; Study 2: *N* = 243) were conducted to close this research gap. The students completed the Intelligence-Structure-Test 2000 R measuring general reasoning ability, and a well-established personality inventory based on the Five Factor Model. Academic achievement was operationalized via Grade Point Average. Using hierarchical regression and moderation analyses, Study 1 revealed that Conscientiousness interacted with intelligence when predicting academic achievement: there was a stronger association between intelligence and academic achievement when students scored higher on the Conscientiousness scale. Study 2 confirmed the findings from Study 1 and also found a moderation effect of Neuroticism (stronger association between intelligence and academic achievement with lower values on the Neuroticism scale). Analyses at the facet level revealed much more differentiated results than did analyses at the domain level, suggesting that investigating personality facets should be preferred over investigating personality domains when predicting academic achievement.

## 1. Introduction

Since the improvement of learning is the central goal of Educational Psychology, the investigation of factors contributing to academic achievement is among its most important issues. Many predictors of academic achievement have already been investigated, but no source of variance has been found to be as strong as the student him- or herself [1]. Among the student predictors, cognitive ability is surely the most important one, setting the stage for what is theoretically possible for the student to achieve [2,3]. However, non-cognitive factors such as personality traits might also exert an influence on academic achievement given their influence on the student’s work approach [4]. More precisely, they might decide upon how well a student manages to convert his or her intelligence into academic achievement, that is, they might interact with intelligence in the prediction of academic achievement. Hitherto, however, only a little attention has been paid to possible interaction effects between personality and intelligence in forming academic achievement. Therefore, in two studies, we aimed to investigate the interaction effects between personality traits and intelligence in the prediction of adolescents’ grade point average (GPA). Moreover, we examined whether the investigation of personality facets instead of broader personality factors in the interaction with intelligence would provide more nuanced results than the investigation of broad factors only. 

### 1.1. Intelligence and Personality as Predictors of Academic Achievement

When it comes to the prediction of academic achievement, there is no doubt that general intelligence (*g*) is the most powerful single predictor [3,5]. This is even true if academic achievement is operationalized via grades instead of academic achievement tests. Correlations between general intelligence and grades most often range from *r* = 0.3 to *r* = 0.5, depending on different factors such as the school subject, years of education, or the selectivity of the sample [2,5,6]. The importance of intelligence notwithstanding, a maximum correlation of *r* = 0.5 means, on the other hand, that the predictive power of intelligence never exceeds 25% variance explained. Therefore, still, other variables must be at work contributing to academic achievement.

After being abandoned for most of the second half of the last century, there has been a renewed interest in personality as a predictor of academic achievement. Especially the Big Five: Neuroticism (N), Extraversion (E), Openness to Experience (O), Agreeableness (A), and Conscientiousness (C) [7], have been scrutinized. N might negatively relate to academic achievement because individuals scoring high on N questionnaires also display higher values on performance anxiety questionnaires, which is, in turn, detrimental to achievement [8]. E and A comprise some characteristics—such as talkativeness (E) or compliance (A)—that might bring out academic behavior that is valued by teachers and leads to better achievement, for example, high engagement and cooperation within learning groups or high participation in class [9]. Students high in O might benefit because they have, on average, higher scores on tests of intelligence, verbal skills, and general knowledge [10]. Furthermore, they use more effective in-depth learning strategies than individuals low in O [11]. Finally, C should be related to academic achievement, because students scoring high on C questionnaires are stronger oriented toward achievement and more ambitious, better organized, more reliable, and more self-disciplined [12,13].

In the meantime, a number of studies have mostly established small but nevertheless non-trivial correlations between certain personality dimensions and grades [12,14,15,16,17,18,19,20,21]. Taking the emerging evidence together, Poropat [22] revealed in his meta-analysis, small but consistent correlations between grades and some of the Big Five. The strongest correlation was found for C (*r* = 0.19), followed by O (*r* = 0.10) and A (*r* = 0.07), whereas there were no correlations with N and E. At least similar results were reported by O’Connor and Paunonen [9]. In another meta-analysis investigating the role of the Big Three, Poropat [23] noted significant but (very) small correlations between academic achievement and Psychoticism (*r* = −0.06), N (*r* = −0.06), and E (*r* = 0.02). 

Importantly, the correlations between academic achievement and personality (especially C) still held when intelligence was controlled for [20,24,25,26,27,28]. This seems even to be true if academic achievement is operationalized via a standardized academic achievement test [29] and if personality dimensions are other-rated instead of self-rated [30]. Thus, both personality and intelligence play an important, unique role in the prediction of academic achievement. Therefore, theoretical models seeking to explain academic achievement should consider both personality and intelligence.

To sum up, there have been consistent correlations found for some personality traits, whereas there were zero correlations for other personality traits. However, zero correlations do not necessarily mean that those personality traits are not important for academic achievement. O’Connor and Paunonen [9] argued that investigating personality facets instead of broad personality domains might give a clearer picture of the association between personality and academic achievement [31,32]. In two studies with university students, Paunonen and Ashton [33,34] found support for this claim: Achievement, Understanding, and Play as facets of C, O, and E, respectively, predicted GPA (0.17 ≤ |*r*| ≤ 0.26). When the facets were combined with the respective other facets of the broader personality factors, these factors provided a weaker prediction of GPA in both studies (0.01 ≤ |*r*| ≤ 0.21). Therefore, even though broad traits might seem unrelated to academic achievement, at first sight, certain facets of these personality traits might still be very useful in the prediction of academic achievement.

### 1.2. The Interaction between Intelligence and Personality in Predicting Academic Achievement

Some of the models predicting academic achievement assume that intelligence and personality predict academic achievement independently of each other, that is, both constructs’ contribution to academic achievement is seen as additive [35]. However, older theories predicting performance from intelligence and motivation suggested that both constructs should interact with each other in an ordinal manner [36,37]. More precisely, they stated that on the one hand, the association between performance and intelligence should be moderated by motivation. If motivation is low, intelligence should matter less for performance than when motivation is high because smart individuals with low motivation might waste their intellectual abilities instead of investing it in performance. On the other hand, the association between performance and motivation should be moderated by intelligence. If intelligence is low, motivation should matter less for performance than when intelligence is high, because even high motivation might be futile in the face of missing ability [38]. 

Since motivation and personality are related [12,39,40], there is the hypothesis that performance is an interactive function of intelligence and personality, as well [41,42]. Whereas intelligence describes the limiting conditions academic performance depends on [2], personality—just like motivation—describes how one approaches a task [43]. Therefore, personality might moderate the relationship between intelligence and academic achievement (or intelligence might moderate the relation between personality and academic achievement) [41]. Following traditional performance theories, it should be especially likely to find an interaction between intelligence and personality traits that are related to achievement motivation.

Studies investigating the interaction between intelligence and personality in the prediction of performance mainly focused on C or its facet Achievement Striving, due to their close relation to achievement motivation. Rather mixed results were found for C: Di Domenico and Fournier [24], as well as Ziegler et al. [21], found that C interacted with intelligence in the prediction of GPA. The higher C was, the higher the relation between intelligence and GPA, suggesting that students scoring high on C questionnaires might make their cognitive ability more fruitful through hard and accurate work than students scoring low on C questionnaires. On the other hand, studies predicting job performance did not find such an interaction [38,44,45].

The results concerning Achievement Striving, however, seem to be more homogenous. Most studies found a significant interaction between Achievement Striving and intelligence when predicting academic achievement [21,42] (in the study by Ziegler et al. [21], this interaction effect was only found when the sample was split according to academic achievement. There was an ordinal interaction effect in high performers and a rather semi-ordinal interaction effect in low performers, canceling each other out when regarding the entire sample). Other facets of C such as Competence, Dutifulness, and Self-Discipline are also related to achievement motivation [12,46,47] and might therefore also interact with intelligence in the prediction of academic achievement.

Not only C and its facets, but also the other Big Five dimensions and their facets are related to achievement motivation [40,46]. Therefore, they might also interact with intelligence in the prediction of academic achievement. However, an explicit prediction with regard to an interaction of ability and a Big Five personality domain (besides C) or facet has only been made with regard to N [48,49] and O, but this has rarely been investigated. Zhang and Ziegler [50] found that there was an association between figural reasoning and academic achievement only for students with average and below average scores on the O questionnaire. However, figural reasoning did not matter for academic achievement when O scores were above average, indicating a buffer effect of high O against low figural reasoning ability. Similar results were found by Ziegler et al. [51] for O and fluid intelligence when predicting vocabulary. Heaven and Ciarrochi [18] found that O to ideas (Intellect) among 7th graders only marginally predicted GPA 3 years later, but there was a significant interaction between Intellect and intelligence: The association between Intellect and GPA was stronger for students with higher scores on the intelligence test (+0.5 *SD*). For students with lower scores on the intelligence test (−0.5 *SD*), there was no association. 

However, as studies concerning the other Big Five than C are scarce, there is a further need for studies investigating the joint effect of personality domains (besides C) and intelligence that also consider a possible interaction between the personality domain and intelligence. Moreover, possible interaction effects between the personality facets and intelligence in the prediction of academic achievement have not sufficiently been clarified yet. It might be that possible interactions between broad personality factors and intelligence do not apply to all facets related to the respective broad factor. At the same time, some facets might exhibit much stronger interaction effects with intelligence than the broad factor.

### 1.3. Aims of the Present Investigation

The aims of our investigation were twofold and, therefore, we conducted two studies. As a first step, we investigated the possible interaction effects between intelligence and the Big Five in predicting academic achievement (Study 1). We expected an interaction effect between intelligence and C. However, we did not limit our investigation to C, but also expanded our view to the other Big Five dimensions, which have so far been somewhat neglected as compared to C. Due to their relation to achievement motivation, they might interact with intelligence in the prediction of academic achievement, as well. In Study 2, we first tested the replicability of the results from Study 1. Then, we investigated whether the inspection of personality facets (as opposed to broad personality factors) in interaction with intelligence provided more differentiated results when predicting academic achievement.

## 2. Study 1

### 2.1. Materials and Methods 

#### 2.1.1. Sample and Procedure 

*N* = 421 students (*M*_age_ = 16.43 years, *SD*_age_ = 0.55; 208 girls) from 5 different high schools (Gymnasiums) in Germany took part. The Gymnasium is the highest track in Germany’s secondary school system. It leads to the Abitur, which allows for university enrollment. All students attended 11th grade. The students can be considered as the typical population of the Gymnasium in Germany (that is, the majority being Caucasian from medium to high socioeconomic status homes).

Testing took place during a regular school day and was conducted in groups of about 20 students. Trained students and research assistants administered the tests according to standardized instructions. Participation was voluntary, and students were allowed to take part only if their parents had completed written consent forms.

#### 2.1.2. Measures 

*Intelligence*. We administered the basic module of the Intelligence-Structure-Test 2000 R [52]. The IST 2000 R basic module measures reasoning ability using verbal, numerical, and figural material. The composite score out of these 3 domains indicates general reasoning ability, which is very closely related to general intelligence (*g*). Therefore, it can be used as a proxy for general intelligence.

*Personality*. We assessed the Big Five (Neuroticism, Extraversion, Openness to Experience, Agreeableness, and Conscientiousness) with the German version of the NEO-FFI [53]. This instrument consists of 60 items, covering each of the Big Five with 12 items. Answers on the items ranged from 0 (*strong disagreement*) to 4 (*strong agreement*). Reliabilities in the present study were α = 0.85 for N, α = 0.78 for E, α = 0.68 for O, α = 0.77 for A, and α = 0.85 for C.

*Academic achievement*. To operationalize academic achievement, we used the Grade Point Averages (GPAs) from the students’ report cards obtained 3 months after testing. The report cards were provided by the schools. In Germany, grades range from 1 (outstanding performance) to 6 (complete failure).

The data set and the code book for Study 1 can be obtained as described in the section “Supplementary Materials”.

#### 2.1.3. Analyses 

We first inspected descriptive statistics and intercorrelations for all variables under study. We then performed a hierarchical regression analysis for every personality trait separately, using SPSS 25.0. To this end, we centered both intelligence and the respective personality trait before entering them into the regression model. First, we predicted GPA from intelligence only (Step 1). In Step 2, we added each of the Big Five separately to inspect their incremental predictive value above and beyond intelligence. Finally, we added the interaction term between intelligence and the respective personality trait (product of the centered predictors) to test for a possible interaction effect (Step 3). If the interaction effect was statistically significant, we performed a simple slope analysis using the program “Interaction”, version 1.7.2211 [54]. Additionally, we calculated confidence bands and regions of significance to determine at which moderator levels the association between intelligence and GPA became statistically significant (α = 0.05) or insignificant, respectively [55].

### 2.2. Results

#### 2.2.1. Descriptive Statistics and Intercorrelations 

Table 1 depicts the descriptive statistics for each variable of Study 1 and their intercorrelations. In order to compare our sample with the norm samples of the instruments, we also calculated the *T* values (*M* = 50, *SD* = 10) and the standard deviations of the *T* values (for the personality factors, the norm samples for 16- to 20-year old men and women, respectively, from 2007 [56] were used, because there is no standardization for the German NEO-FFI edition from 1993; the instrument itself remained unchanged from 1993 to 2007). From the *T* values, it can be seen that the sample was slightly above average, and showed some variance restriction, in intelligence, which is typical for students from Gymnasiums [57,58,59]. Regarding personality, all the factors but O matched the mean of the norming samples quite well and did not show any greater restriction in variance. The correlations were mostly in line with the findings from the previous research. Intelligence showed the strongest correlation with GPA (*r* = −0.31, *p* < 0.001), followed by C (*r* = −0.27, *p* < 0.001), and O (*r* = −0.16, *p* < 0.01). The other Big Five dimensions did not significantly correlate with GPA. The associations were also roughly in line with what is known about the correlations between the Big Five and intelligence [60].

#### 2.2.2. Hierarchical Regression and Moderation Analyses

Our central research question in Study 1 was whether the Big Five would interact with intelligence in the prediction of academic achievement. To answer this question, we performed a hierarchical regression analysis for every personality trait as described in Section 2.1.3. The detailed results are exemplarily provided for C in Table 2.

**Figure 1:**
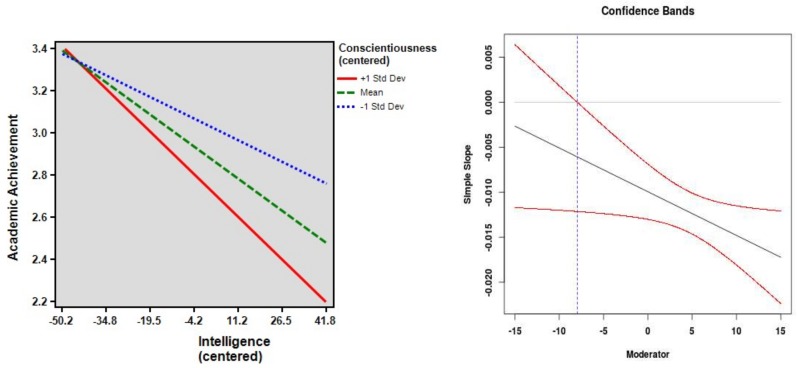
The regression of academic achievement (GPA) on intelligence at high, mean, and low scores on Conscientiousness (on the **left**) as well as confidence bands and region of significance (on the **right**). The dotted line marks the score of the moderator at which the association between GPA and intelligence becomes statistically significant (*p* < 0.05).

C predicted the GPA above and beyond intelligence (β = −0.25, *p* < 0.001). It also interacted with intelligence in predicting GPA (β_IA_ = −0.10, *p* = 0.03). The interaction term explained an additional 1% of the GPA variance (Δ*R*^2^ = 0.009). The simple slope analysis revealed that the association between intelligence and GPA was stronger when C was higher (see Figure 1, left side). In the group with higher scores on C (+1 *SD*), there was a strong association between intelligence and GPA (*t* = −6.19, *p* < 0.001). In the group with lower scores on C (−1 *SD*), the association between intelligence and GPA was weaker (*t* = −3.03, *p* = 0.003). The centered C scores ranged from −17.04 to 17.96. The region of significance began at a C score of −7.97 (see Figure 1, right side). This means that for students who displayed higher scores on C than −7.97, the correlation between intelligence and GPA became statistically significant (*p* < 0.05).

O predicted the GPA above and beyond intelligence (β = −0.13, *p* = 0.005) but did not interact with intelligence in the prediction of GPA. As could be expected from their very low correlations with GPA (see Table 1), the other Big Five dimensions N, E, and A did not predict GPA incrementally. At least for A, however, there was a small interaction effect with intelligence (β_IA_ = −0.08, *p* = 0.09, Δ*R*^2^ = 0.006), indicating a stronger association between intelligence and GPA when scores on A were high (*t* = −5.69, *p* < 0.001) than when scores on A were low (*t* = −3.77, *p* < 0.001). 

### 2.3. Discussion Study 1

Taking the results together, Study 1 showed, first, that at least C and O provided an increment in the prediction of GPA above and beyond intelligence. This is in line with previous research (see Section 1.1). Second, apart from these main effects, students with higher scores on C showed stronger associations between intelligence and GPA than students with lower scores on C. This is in line with the findings from Di Domenico and Fournier [24] and Ziegler et al. [21], but not in line with studies investigating such an interaction effect when predicting job performance. Students scoring high on A showed somewhat stronger associations between intelligence and GPA than students scoring low on A, but this effect was marginal. Regarding the other Big Five dimensions, there were no significant main or interaction effects. These results might, however, change when taking a closer look at the facet level. Therefore, we conducted another study (Study 2).

## 3. Study 2

### 3.1. Materials and Methods 

#### 3.1.1. Sample and Procedure 

The sample of Study 2 consisted of *N* = 243 11th graders from 3 Gymnasiums (*M*_age_ = 16.53 years, *SD*_age_ = 0.57). The schools were located in two mid-sized towns and in one small town, respectively. The students can, again, be considered as a typical student population of the Gymnasium. The majority (*n* = 134) of the students were male. However, the sex distribution did not significantly deviate from what would be expected in a representative population in the Gymnasium (*χ*^2^ = 3.29, *p* = 0.07). The procedure during data collection was comparable to Study 1.

#### 3.1.2. Measures 

*Intelligence*. As in Study 1, we administered the basic module of the Intelligence-Structure-Test 2000 R [52] to achieve a valid proxy for general intelligence.

*Personality*. We used the German version of the NEO-PI-R [61] to assess the Big Five personality traits and their facets. The NEO-PI-R consists of 240 items, covering each of the Big Five dimensions with 48 items. Each broad trait consists of 6 facets, each measured by 8 items. The items were answered on a five-point Likert scale, ranging from 0 (*strongly disagree*) to 4 (*strongly agree*). The reliabilities in the present study ranged from α = 0.85 to α = 0.91 for the broad factors and from α = 0.49 to α = 0.85 for the facets (see Table 3).

*Academic achievement*. Academic achievement was operationalized the same way as in Study 1. Again, lower values indicated better performance.

The data set and the code book for Study 2 can be obtained as described in the section “Supplementary Materials”.

#### 3.1.3. Analyses 

All analyses were conducted comparably to Study 1. For details, see Section 2.1.3.

### 3.2. Results

#### 3.2.1. Descriptive Statistics and Intercorrelations 

In Study 2, we first aimed to test the replicability of the findings from Study 1. The central research question of Study 2, however, was whether narrow personality facets would yield more differentiated results than broad personality traits in the interaction with intelligence when predicting academic achievement. 

Descriptive statistics for each variable of Study 2 as well as their intercorrelations are shown in Table 3. Comparably to Study 1, the sample was above average, and showed some variance restriction, in intelligence. All personality domains but O showed means that were comparable to the norm sample. N, O, and C showed slight degrees of variance restriction. Of the facets, most means were comparable to the norm sample (with some notable deviations only for E-Excitement-Seeking, O-Aesthetics, O-Feelings, and O-Values). Eleven of the 30 facets also showed some restriction in variance. As in Study 1, intelligence showed the strongest correlation with GPA (*r* = −0.33, *p* < 0.001), but there were also some notable correlations between personality and GPA. In the following, we will report the results for each domain and its facets in a separate paragraph.

#### 3.2.2. Neuroticism and Its Facets

**Figure 2:**
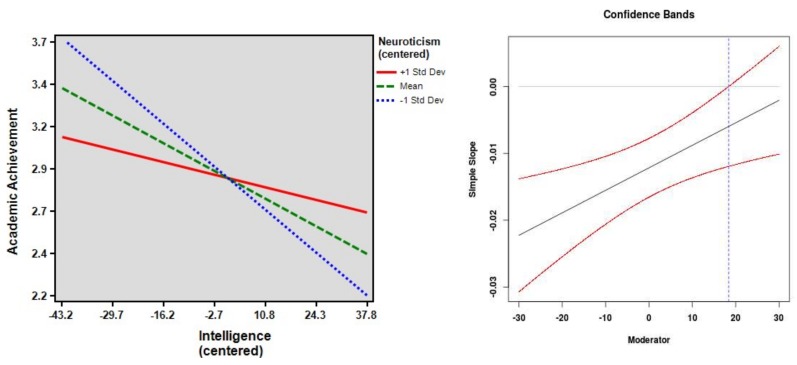
The regression of academic achievement (GPA) on intelligence at high, mean, and low scores on Neuroticism (on the **left**) as well as the confidence bands and region of significance (on the **right**). The dotted line marks the score of the moderator at which the association between GPA and intelligence becomes statistically significant (*p* < 0.05).

Neither N (*r* = −0.01) nor any of its facets (0.02 ≤ |*r*| ≤ 0.04) were correlated with GPA (see Table 3). Therefore, there was no incremental prediction of GPA above and beyond intelligence from N or its facets. However, there were some interactions of N and its facets with intelligence when predicting GPA. The association between intelligence and GPA was stronger when students scored lower on N (β_IA_ = 0.18, *p* = 0.004, Δ*R*^2^ = 0.032; see Figure 2, left side). As can be seen from Figure 2 (right side), the association between intelligence and GPA was statistically significant when N values were lower than 18.37. It was also stronger when students scored lower on the facets Vulnerability (β_IA_ = 0.19, *p* = 0.003, Δ*R*^2^ = 0.034), Impulsiveness (β_IA_ = 0.18, *p* = 0.006, Δ*R*^2^ = 0.030), and Angry Hostility (β_IA_ = 0.14, *p* = 0.025, Δ*R*^2^ = 0.020). Similar results occurred for Depression (β_IA_ = 0.12, *p* = 0.05, Δ*R*^2^ = 0.015) and Anxiety (β_IA_ = 0.12, *p* = 0.06, Δ*R*^2^ = 0.014), although statistical significance was narrowly missed. There was no interaction effect between intelligence and the facet Self-Consciousness.

#### 3.2.3. Extraversion and Its Facets

There were at most small but no statistically significant correlations between E (*r* = 0.01) or its facets (0.03 ≤ |*r*| ≤ 0.12) and GPA, and there were no interaction effects between E nor its facets with intelligence when predicting GPA.

#### 3.2.4. Openness and Its Facets

There were some notable findings with regard to O and its facets: O was significantly correlated with GPA (*r* = −0.20, *p* = 0.002), and so were Ideas (*r* = −0.25, *p* < 0.001) and Aesthetics (*r* = −0.14, *p* = 0.028), but not the other facets (Actions, Fantasy, Feelings, and Values; −0.04 ≤ *r* ≤ −0.11). When intelligence was controlled for, O (β = −0.16, *p* = 0.009), Ideas (β = −0.18, *p* = 0.005), and Aesthetics (β = −0.14, *p* = 0.023) still predicted GPA. However, they did not interact with intelligence. Instead, there was an interaction between intelligence and Actions: Intelligence was associated stronger with GPA when students scored lower on Actions (β_IA_ = 0.14, *p* = 0.024, Δ*R*^2^ = 0.020).

#### 3.2.5. Agreeableness and Its Facets

The correlation between A and GPA (*r* = −0.10) was not statistically significant, but it was in the same range as found by Poropat [22]. Two of the facets significantly correlated with GPA, namely Tender-Mindedness (*r* = −0.20, *p* = 0.003) and Straightforwardness (*r* = −0.15, *p* = 0.026). The correlations between the other facets (Altruism, Compliance, Modesty, and Trust) and GPA ranged from *r* = −0.10 to *r* = 0.06 (n.s.). When controlling for intelligence, slight suppression effects became apparent: the predictive value of A (β = −0.13, *p* = 0.035), Tender-Mindedness (β = −0.22, *p* < 0.001), and Straightforwardness (β = −0.16, *p* = 0.008) slightly increased, as was the case for Trust, which after controlling for intelligence narrowly reached statistical significance (β = −0.13, *p* = 0.041). Although the interaction effect between A and intelligence in predicting GPA was not statistically significant, it was comparable to the effect observed in Study 1 (β_IA_ = −0.09, *p* = 0.171, Δ*R*^2^ = 0.007). Of the facets, Compliance (β_IA_ = −0.16, *p* = 0.009, Δ*R*^2^ = 0.027) and Altruism (β_IA_ = −0.13, *p* = 0.043, Δ*R*^2^ = 0.016) displayed interaction effects with intelligence. The association between intelligence and GPA was stronger when students scored higher on A (marginal), Compliance, and Altruism, respectively.

#### 3.2.6. Conscientiousness and Its Facets

Again, C showed the strongest associations with GPA of all Big Five. Correlations with GPA were *r* = −0.20 (*p* = 0.002) for C and comparable in size for most of its facets (Dutifulness: *r* = −0.23, *p* < 0.001; Self-Discipline: *r* = −0.22, *p* = 0.001; Achievement Striving: *r* = −0.20, *p* = 0.003; Competence: *r* = −0.14, *p* = 0.035). It was not significant for Deliberation (*r* = −0.11) and Order (*r* = −0.01). When controlling for intelligence, nearly all of the significant predictions held (C: *r* = −0.19, *p* = 0.002; Dutifulness: *r* = −0.24, *p* < 0.001; Self-Discipline: *r* = −0.20, *p* = 0.001; Achievement Striving: *r* = −0.18, *p* = 0.004; Competence: *r* = −0.12, *p* = 0.064). C and all facets except from Order significantly interacted with intelligence: Intelligence was more predictive of GPA when students scored higher on C (β_IA_ = −0.18, *p* = 0.004, Δ*R*^2^ = 0.031), Competence (β_IA_ = −0.14, *p* = 0.026, Δ*R*^2^ = 0.019), Dutifulness (β_IA_ = −0.16, *p* = 0.010, Δ*R*^2^ = 0.024), Achievement Striving (β_IA_ = −0.13, *p* = 0.040, Δ*R*^2^ = 0.016), Self-Discipline (β_IA_ = −0.14, *p* = 0.020, Δ*R*^2^ = 0.020), and Deliberation (β_IA_ = −0.18, *p* = 0.004, Δ*R*^2^ = 0.031).

Table 4 provides an overview of the statistically significant results from Study 2. Table 4 also comprises the regions of significance of the association between intelligence and GPA. It also presents the minimum and maximum values of the centered moderator scores in order to facilitate the classification of the regions of significance.

### 3.3. Discussion Study 2

To sum up, with regard to the broad domains, we found C and N to interact with intelligence in the prediction of academic achievement. The interaction of A was statistically marginal, but consistent with Study 1. With regard to the facets, most interaction effects we found referred to facets which belonged to a broad domain for which we had found an interaction effect, as well. However, of those domains, only some facets showed an interaction effect whereas others did not. In some cases (especially for O and A), their effects were stronger than the effect of the respective domain, whereas in other cases, they were slightly smaller (or equal). These findings suggest that using personality facets in the prediction of GPA might provide more nuanced results than relying on broad personality domains.

## 4. General Discussion

Some personality traits have been proven to predict academic achievement above and beyond intelligence. However, their interaction with intelligence when predicting academic achievement has long been neglected and the relevant studies have mainly been limited to Conscientiousness (C). Furthermore, there is still research required with regard to the question whether it is sufficient to rely on broad personality traits or whether it might be worthwhile investigating the interaction effects at the facet level. Therefore, in the two studies we expanded our view to all the Big Five personality domains and tested their interactions with intelligence when predicting students’ GPAs. In Study 2, we also tested the interaction effects of the domains’ facets.

### 4.1. Interaction Effects between Intelligence and Broad Personality Domains 

Taking the results of both studies together, we found that only C clearly and consistently interacted with intelligence when predicting academic achievement. The high predictive value of intelligence for academic achievement is unequivocal [3,5]. We found—in accordance with previous studies also using GPA as the criterion variable [21,24]—that the predictive value of intelligence is even higher when students display higher scores on C. Students with high C might work especially thoroughly and hard [62,63], which might enable them to use their full cognitive potential. This might pay off, especially for students with high intelligence, receiving excellent grades, whereas students with high intelligence, but low C, might diminish their opportunities to receive excellent grades because of their working style being rather careless and sloppy. However, working style might be less decisive for academic achievement of students with low intelligence, because even thorough and hard work might compensate for cognitive deficits only to a very limited extent. 

Whereas we found no interaction between Neuroticism (N) and intelligence in Study 1, we found in Study 2 that among students with lower scores on N, there was a stronger relation between intelligence and GPA than among students with higher scores on N. The difference in results between Study 1 and Study 2 might be explained by the fact that N was assessed more comprehensively in Study 2 than in Study 1, using the NEO-PI-R instead of the NEO-FFI. The found effect in Study 2 is in line with the proposition by Zeidner [49] that N should lower the correlation between intelligence and achievement because a high N should hamper highly-abled students from fulfilling their potential, whereas N should be less decisive for students with low ability. 

As became apparent, however, the interaction effect in Study 2 was semi-ordinal, that is, high N was not always detrimental to students’ grades, but partly even advantageous: Among students with lower intelligence test scores, those with higher scores on N had better grades than those with lower scores on N (whereas it was the expected to be the other way around for students with higher intelligence test scores). Against this background, it is interesting to note that there are sometimes positive correlations found between N and academic achievement [64]. 

How can it be explained that N seems to work differently for students with different ability levels? The most often negative correlation found between N and academic achievement is usually explained by performance anxiety and its negative effects on performance [8,65]. The cognitive component “worry” is especially detrimental to achievement, because it requires cognitive capacities so that these capacities cannot be devoted to the task anymore [66,67]. Importantly, performance anxiety is especially impeding when students work on difficult tasks [8,49], possibly because the cognitive capacities occupied by worry would be badly needed for these tasks. Difficult tasks in a test, in turn, have a high discriminatory power at the upper end of the grading scale. Simply put, solving or not solving very difficult tasks in a test might decide upon whether a highly-abled student receives an A or a B. Thus, a highly-abled student with a high N (high impediment by worry) will probably receive the B, whereas a highly-abled student with a low N (no impediment by worry) will probably receive the A. 

However, for a student with low ability, the question would rather be whether he or she receives, say, a D or an E. Tasks with discriminatory power in this range of the grading scale will be much easier and probably better to solve by memorization than by thinking. That is where the beneficial side of performance anxiety might come into play: Performance anxiety can motivate students to prepare for a test extensively (not to say excessively) because their primary objective is to avoid failure [8]. In doing so, students with high-performance anxiety tend to prefer surface learning strategies over elaboration in order to avoid gaps in knowledge [8], but this approach should be sufficient (or even particularly useful) for solving tasks with low difficulty. Furthermore, the performance anxiety component “emotionality” increases the physiological arousal, which is—at least to a certain extent—useful when working on rather easy tasks [8,68]. Therefore, a low-ability student with high N might prepare extensively for the test, have an optimal arousal level during the test, and finally pass it with a D, whereas a low-ability student with low N might take the preparation for the test (too) lightly, have a (too) low arousal level during the test, and finally fail it. Future studies should test the semi-ordinal interaction effect for its replicability. However, there already is support coming from the study by Sung, Chao, and Tseng [69]. In this study among 9th graders with above-average scores on a standardized academic achievement test, there was a negative correlation between performance anxiety and academic achievement, whereas, among students with below-average scores, there was a positive correlation between both constructs. Ziegler et al. [21] also found that the moderation effects of personality (in their case, Achievement Striving) depend on the performance level. In line with this finding, LaHuis et al. [70] found a quadratic relation between Conscientiousness and job performance. Therefore, Ziegler et al. [21] suggested that future studies should pay more attention to the performance level when investigating interaction effects of personality and intelligence in the prediction of performance. Our findings for N lead to the same recommendation.

In contradiction to the study by Zhang and Ziegler [50], Openness (O) did not moderate the relation between intelligence and academic achievement. Age differences between the samples appear rather unlikely as a valid explanation for this discrepancy because the age difference was only 1 year. It might be due to cultural differences, since the sample of Zhang and Ziegler [50] consisted of Chinese students, whereas our sample was German. On the other hand, Ziegler et al. [51] investigated adolescents and psychology students from Germany, too, and found an interaction effect between O and intelligence when predicting vocabulary. Maybe the selectivity of our samples (students from the Gymnasium) hindered us from finding the moderating effect of O. Indeed, there was some variance restriction in O at least in Study 2. 

Additionally, the role of Agreeableness (A) as a moderator of the relation between intelligence and academic achievement seems notable. Although its interaction with intelligence remained statistically non-significant, we replicated it in both size and direction. Whereas in Study 1, the interaction was semi-ordinal, it was ordinal in Study 2. In Study 2, A did not matter for students with low ability, but it did matter for students with high ability: students with high A received better grades than students with low A. It is possible that when teachers have to decide which grade at the high end of the grading scale a student gets, A might impact their judgment. A likely influences students’ working behavior, for example, their cooperation in class and in group work, which might create a positive image of the student in the teacher’s view. This positive image could, in turn, influence the teacher’s judgment of the student’s academic ability in terms of a halo effect [71,72]. Since grading also has the function of disciplining students [73], it is also possible that the teacher deliberately awards desirable behavior with good grades. However, if this explanation holds true, then why is this not also the case for students with low ability? In Study 1, high A was even detrimental to academic achievement among low-ability students. Possibly, A has a differential impact on students’ behavior, depending on their intelligence. As conjectured above, A might cause desirable learning behavior in students with high intelligence. However, as lower intelligence is accompanied by lower behavioral engagement in school [74], students with low intelligence might “invest” their A rather in cooperative behavior that is not related to learning and might even disturb the lessons, for example, activities with friends during the lessons. Those behaviors would probably not be valued by the teacher and they are not beneficial for learning, either, both of which would result in worse grades. However, as the interaction effect was not statistically significant in both studies, one should not over-interpret it. Future studies should further investigate this effect using larger sample sizes before final conclusions can be drawn. 

However, it already seems that certain facets of A are more important than A as a whole, as A comprises some facets which showed a stronger interaction with intelligence than A itself. This already points to the assumption that it might be worthwhile to go down to the facet level when investigating the interaction between intelligence and personality in predicting academic achievement.

### 4.2. Interaction Effects between Intelligence and Narrow Personality Facets 

We mostly found significant interaction effects at the facet level if the respective trait had displayed an interaction effect. However, the investigation of interaction effects at the facet level revealed more nuanced results than the analyses at the domain level: not every facet of a certain domain interacted with intelligence and some facets showed greater interaction effects than the domain itself. Thus, facets give us a clearer picture of how personality and intelligence interact in predicting academic achievement. More precisely, taking a look at the facet level gives us insights into which facets are the driving factors behind an interaction effect at the domain level. 

With regard to N, the interaction effect was mainly due to Vulnerability, Impulsiveness, and Angry Hostility. Depression and Anxiety contributed to a somewhat more limited degree, whereas Self-Consciousness did not contribute to the interaction effect of N. This finding is interesting, as the line of reasoning made above about the interaction effect of N primarily referred to anxiety as the driving factor behind the interaction effect. Anxiety indeed plays a role, but there seem also to be other facets of N at work. Interestingly, the facets’ interaction effects with intelligence were semi-ordinal. Among students with high ability, the facets impacted grades negatively as could be expected. However, among students with low ability, high values in these facets were beneficial for academic achievement. 

The findings were even more nuanced with regard to O and A. Whereas effects at the domain level were either modest (A) or even non-existent (O), analyses at the facet level showed that A and O were nevertheless relevant after a sort: interactions between intelligence and Compliance, Altruism, and O to Actions, respectively, were statistically and practically significant (and the nature of the interaction effects was again semi-ordinal). Against this background, it should again be remembered that grades do not only reflect a student’s ability but also to some degree the student’s behavior as perceived by the teacher. Some facets (such as Compliance, Altruism, and O to Actions) might be viewed by the teacher as especially relevant behavior in the classroom and might, therefore, exhibit a greater influence on grade assignments than other facets (for example, Modesty, O to Fantasy, Feelings, or Values). 

For C, then again, nearly all facets (Competence, Dutifulness, Achievement Striving, Self-Discipline, Deliberation), except Order, contributed to a roughly equal extent to the interaction effect at the domain level, indicating no great difference in the results between the domain and the facet level. Thus, nearly all facets of C seem to be important for academic achievement. Comparable to Study 1, most interaction effects were rather ordinal in nature: when students had higher scores on the intelligence test, C and its facets mattered. When students had lower intelligence test scores, they did not. This is largely in line with previous research on Achievement Striving [21,42]. However, whereas Ziegler et al. [21] found interaction effects only when they split up the data set according to achievement level, we found an interaction effect across the entire sample. In so doing, we confirmed the assumption that also the other facets of C should show interaction effects because of their relation to achievement motivation [12,46,47,75]. It might be interesting for future studies to examine whether C and its facets would still interact with intelligence in the prediction of academic achievement if achievement motivation was controlled for. Although Steinmayr and Spinath [12] found that C did not predict GPA above and beyond the need for achievement, our findings suggest that achievement motivation might not be the only cause of C’s interaction with intelligence, given the interaction effect found for Deliberation. Interestingly, Deliberation provided the only semi-ordinal interaction effect of C, suggesting that this facet might indeed work differently from the other facets. 

Taken together, we found that (1) C consistently interacts with intelligence in the prediction of academic achievement, but that (2) taking a closer look at the facet level tells us that the other Big Five (except E) are in part also relevant. Thus, investigations at the facet level provide us with more detailed and nuanced information on the interaction between personality and intelligence when predicting academic achievement. 

### 4.3. Limitations and Future Directions 

Although our results are in favor of a more in-depth view of personality traits, one caveat should be noted, as it might also be relevant for future studies: the comparability of the results from different hierarchical levels, such as broad domains versus narrow facets, is limited. Typically, the reliabilities of facet scales are lower than those of the domain scales. This was also the case in our study. Therefore, the chance to detect effects at the facet level is smaller than at the domain level. This might lower the chance also for future studies to replicate facet level effects. Accordingly, the facet effects in our study might somewhat underestimate the “true” facet effects. Therefore, the future studies might use analyses with latent variables to exclude the measurement error.

One point that might also have suppressed the effects to at least some extent is the variance restriction in some of the variables. Especially in Study 2, there was a variance restriction in N, O, and C as well as in some of the personality facets. There was also some variance restriction in intelligence, which might explain why the correlation between intelligence and academic achievement was among the lower bound of correlations usually reported in other studies. Thus, the found effects can probably be seen as lower bound estimates. In the case of O, the variance restriction might even have hindered us from identifying any effect (see above).

Another important note is that we used grades as an operationalization of academic achievement. As already noted, grades do not only reflect ability aspects (for example, did you correctly solve problems in the exam? Did you give the right answers?), but also non-cognitive aspects (for example, did you improve during the term? Did you show commitment to do so?). Thus, grades might be a better criterion to detect interactions between intelligence and personality than the pure ability criteria such as scholastic achievement tests. This might also explain why studies predicting job performance did not find any interactions between personality and intelligence [44,45]. Future studies might, therefore, compare the predictability of grades and scholastic achievement tests by interactions of personality and intelligence. They might also consider other criteria that comprise even stronger non-cognitive components than do grades [2]. In this case, we would expect that interaction effects would be even stronger than in our study.

Importantly, the impact of ability on our grades becomes weaker and the impact of personality becomes stronger during school time [76]. Whereas Lievens et al. [76] found this result for medical school, it might also apply to primary and secondary education. Therefore, longitudinal studies or cross-sequential studies focusing on different age groups might find different results than we did for adolescents. For elementary school children, for example, interaction effects between personality and intelligence might be weaker, whereas, for older students than those used in the present study, they might be stronger.

## 5. Conclusions 

Despite these limitations, our study showed that broad personality traits, and among them especially C, as well as some of their facets, interact with intelligence in the prediction of academic achievement as measured by GPA. Paying special attention to the facet level brings more differentiated and nuanced results to light than relying on the broad personality traits only. In this respect, not only C, but also parts of N, O, and A interact with intelligence. All in all, the interaction effects were numerous. Therefore, future theoretical and practical endeavors to explain academic achievement should more strongly integrate non-linear relations between personality and academic achievement.

## Figures and Tables

**Table 1:** The means (*M*), standard deviations (*SD*), *T* values, the standard deviation of *T* values, and intercorrelations for intelligence, the Big Five personality dimensions, and grade point average (GPA).

Variable	*M*	*SD*	*T*	*SD_T_*	1	2	3	4	5	6
Intelligence	109.90	16.93	57.65	8.65	–					
Neuroticism	21.00	7.63	47.53	9.25	−0.12 *	–				
Extraversion	30.45	6.46	51.96	9.88	−0.07	−0.30 ***	–			
Openness	27.03	6.68	42.51	9.74	0.11 *	0.10 *	−0.08	–		
Agreeableness	30.25	5.95	51.52	10.05	−0.12 *	−0.16 **	0.27 ***	−0.12 *	–	
Conscientiousness	29.04	6.64	49.77	8.93	0.06	−0.21 ***	0.01	0.05	0.10 *	–
GPA	2.88	0.59	–	–	−0.31 ***	0.08	0.06	−0.16 **	0.03	−0.27 ***

Note. *N* = 408–421. Lower GPA values indicate better performance. * *p* < 0.05; ** *p* < 0.01; *** *p* < 0.001.

**Table 2:** The hierarchical regression of grade point average on centered intelligence and conscientiousness.

Variable	*B*	β	*p*	*R*^2^	Δ*R*^2^
Step 1				0.094	
Intelligence	−0.01	−0.31	<0.001		
Step 2				0.158	0.063 **
Intelligence	−0.01	−0.29	<0.001		
Conscientiousness	−0.27	−0.25	<0.001		
Step 3				0.167	0.009 *
Intelligence	−0.01	−0.29	<0.001		
Conscientiousness	−0.26	−0.25	<0.001		
Intelligence × Conscientiousness	−0.01	−0.10	0.03		

Note. *N* = 408. Lower GPA values indicate better performance. * *p* < 0.05; ** *p* < 0.001.

**Table 3:** The means (*M*), standard deviations (*SD*), reliabilities, and intercorrelations for intelligence, the Big Five personality dimensions, their facets, and grade point average (GPA).

Variable	*M*	*SD*	*T*	*SD_T_*	1	2	3	4	5	6	7	8	9	10	11	12	13	14	15	16	17	18	19	20	21	22	23	24	25	26	27	28	29	30	31	32	33	34	35	36
1 Intelligence	111.70	16.43	58.45	8.44	*0.88*																																			
2 Neuroticism (N)	91.54	19.80	48.45	8.62	−0.05	*0.90*																																		
3 N-Anxiety	15.93	4.63	47.83	8.42	−0.13	0.81	*0.73*																																	
4 N-Angry Hostility	14.93	4.36	49.60	8.95	−0.02	0.69	0.40	*0.70*																																
5 N-Depression	13.69	5.92	48.35	9.87	0.06	0.82	0.62	0.45	*0.85*																															
6 N-Self-Consciousness	16.21	3.91	48.35	8.22	0.02	0.69	0.50	0.30	0.54	*0.57*																														
7 N-Impulsiveness	17.99	4.01	50.70	9.35	−0.01	0.51	0.30	0.35	0.18	0.18	*0.59*																													
8 N-Vulnerability	12.78	4.22	48.99	8.41	0.15	0.83	0.66	0.52	0.61	0.52	0.32	*0.77*																												
9 Extraversion (E)	120.66	19.80	53.47	10.26	−0.10	−0.29	−0.18	−0.16	−0.41	−0.41	0.24	−0.29	*0.91*																											
10 E-Warmth	22.41	4.00	51.58	9.48	−0.11	−0.13	0.03	−0.27	−0.18	−0.19	0.19	−0.11	0.72	*0.77*																										
11 E-Gregariousness	20.65	5.52	52.37	10.13	−0.14	−0.14	−0.04	−0.15	−0.24	−0.28	0.26	−0.10	0.85	0.65	*0.84*																									
12 E-Assertiveness	17.19	4.73	52.56	9.07	−0.01	−0.31	−0.22	0.01	−0.35	−0.44	0.08	−0.38	0.68	0.31	0.45	*0.78*																								
13 E-Activity	17.93	3.38	50.43	8.10	−0.09	−0.25	−0.19	−0.04	−0.31	−0.40	0.17	−0.26	0.66	0.30	0.41	0.50	*0.53*																							
14 E-Excitement-Seeking	20.36	4.41	56.13	9.68	−0.07	−0.20	−0.23	−0.04	−0.25	−0.24	0.14	−0.20	0.66	0.29	0.52	0.34	0.39	*0.60*																						
15 E-Positive Emotions	22.13	5.12	50.06	9.88	−0.04	−0.27	−0.13	−0.22	−0.44	−0.25	0.20	−0.22	0.76	0.59	0.57	0.36	0.40	0.33	*0.82*																					
16 Openness (O)	112.07	16.56	43.78	8.34	0.13	0.12	0.16	0.06	0.10	0.04	0.25	−0.09	0.23	0.26	0.08	0.17	0.22	0.08	0.22	*0.85*																				
17 O-Fantasy	20.03	4.80	46.99	8.51	0.08	0.22	0.17	0.20	0.13	0.14	0.30	0.08	0.09	0.11	−0.01	0.08	0.12	0.03	0.10	0.70	*0.77*																			
18 O-Aesthetics	18.11	5.75	43.93	9.26	0.01	0.20	0.25	0.09	0.15	0.06	0.27	0.05	0.15	0.27	0.06	0.06	0.10	0.00	0.18	0.81	0.49	*0.77*																		
19 O-Feelings	21.01	4.00	45.14	8.21	0.02	0.16	0.22	0.07	0.09	−0.01	0.29	0.03	0.44	0.50	0.34	0.27	0.20	0.17	0.42	0.66	0.43	0.52	*0.74*																	
20 O-Actions	16.88	3.40	47.98	8.22	0.04	−0.21	−0.16	−0.11	−0.21	−0.24	0.05	−0.24	0.37	0.16	0.25	0.28	0.44	0.27	0.25	0.44	0.07	0.24	0.21	*0.49*																
21 O-Ideas	18.60	5.14	47.37	9.35	0.28	−0.02	−0.04	0.01	0.08	0.10	−0.05	−0.22	−0.11	−0.11	−0.21	0.05	0.05	−0.08	−0.14	0.62	0.33	0.36	0.13	0.12	*0.80*															
22 O-Values	17.45	2.88	41.36	7.82	0.03	−0.03	0.05	−0.13	0.05	−0.01	0.00	−0.10	0.03	0.10	−0.01	−0.05	0.00	0.00	0.09	0.43	0.10	0.22	0.15	0.25	0.20	*0.50*														
23 Agreeableness (A)	110.80	17.35	51.06	9.60	−0.10	−0.08	0.07	−0.39	0.01	0.03	−0.11	0.04	0.06	0.36	0.11	−0.20	−0.14	−0.11	0.20	0.04	−0.09	0.09	0.16	−0.03	−0.11	0.20	*0.88*													
24 A-Trust	17.93	4.05	50.53	8.74	−0.08	−0.26	−0.07	−0.41	−0.27	−0.18	−0.02	−0.14	0.37	0.43	0.37	0.07	0.08	0.13	0.45	0.03	−0.14	0.07	0.15	0.10	−0.08	0.07	0.62	*0.71*												
25 A-Straightforwardness	17.56	4.88	51.35	11.06	−0.04	−0.03	0.00	−0.21	0.02	0.05	−0.15	0.13	−0.15	0.06	−0.10	−0.27	−0.16	−0.19	0.00	−0.07	−0.11	−0.03	−0.04	−0.07	−0.11	0.16	0.75	0.30	*0.72*											
26 A-Altruism	21.71	3.65	51.06	9.13	−0.06	−0.15	0.03	−0.36	−0.07	−0.04	−0.06	−0.15	0.28	0.54	0.25	0.03	−0.02	0.05	0.35	0.24	0.07	0.23	0.41	0.05	−0.04	0.22	0.71	0.39	0.35	*0.67*										
27 A-Compliance	15.37	4.26	49.82	9.64	−0.02	−0.19	−0.01	−0.49	−0.06	0.06	−0.29	−0.05	−0.14	0.13	−0.11	−0.25	−0.24	−0.19	0.01	−0.12	−0.18	−0.07	−0.13	−0.13	−0.02	0.12	0.66	0.40	0.40	0.41	*0.65*									
28 A-Modesty	17.68	4.81	51.90	9.97	−0.13	0.19	0.21	−0.08	0.31	0.15	−0.03	0.20	−0.16	0.09	−0.03	−0.31	−0.17	−0.19	−0.11	−0.08	−0.12	0.00	0.04	−0.11	−0.19	0.13	0.70	0.19	0.52	0.34	0.28	*0.77*								
29 A-Tender-Mindedness	20.55	3.53	49.07	9.14	−0.07	0.07	0.10	−0.11	0.04	0.04	0.15	0.08	0.19	0.37	0.16	0.01	0.00	0.04	0.22	0.26	0.18	0.26	0.35	0.06	0.01	0.14	0.69	0.34	0.42	0.54	0.24	0.43	*0.60*							
30 Conscientiousness (C)	108.94	18.00	49.56	8.79	0.07	−0.42	−0.26	−0.24	−0.29	−0.17	−0.43	−0.44	0.10	0.04	−0.05	0.28	0.14	0.03	0.03	0.12	−0.06	0.00	0.03	0.05	0.36	0.04	−0.02	0.07	−0.05	0.13	0.13	−0.26	−0.02	*0.90*						
31 C-Competence	19.92	3.46	49.84	9.17	0.08	−0.47	−0.31	−0.23	−0.40	−0.30	−0.26	−0.52	0.29	0.16	0.10	0.49	0.24	0.11	0.15	0.21	−0.02	0.06	0.18	0.21	0.33	0.04	−0.04	0.09	−0.14	0.22	0.04	−0.29	0.04	0.70	*0.66*					
32 C-Order	17.06	4.56	49.30	9.25	0.00	−0.12	−0.02	−0.02	−0.06	−0.01	−0.32	−0.13	−0.04	−0.04	−0.07	0.09	0.00	−0.07	−0.06	−0.01	−0.04	−0.04	−0.05	−0.13	0.17	−0.01	−0.11	0.00	−0.10	−0.03	0.05	−0.23	−0.11	0.75	0.33	*0.68*				
33 C-Dutifulness	19.71	3.88	48.53	9.01	0.02	−0.30	−0.23	−0.18	−0.22	−0.12	−0.25	−0.32	0.09	0.07	−0.03	0.22	0.10	−0.04	0.08	0.12	0.00	0.02	0.06	0.03	0.27	0.03	0.11	0.11	0.09	0.17	0.14	−0.12	0.08	0.79	0.48	0.47	*0.67*			
34 C-Achievement Striving	18.91	3.97	49.91	8.40	0.06	−0.18	−0.11	0.02	−0.21	−0.12	−0.10	−0.27	0.18	0.01	−0.01	0.29	0.29	0.16	0.08	0.19	0.07	0.06	0.06	0.14	0.29	0.06	−0.19	−0.04	−0.17	−0.02	−0.13	−0.32	−0.04	0.71	0.45	0.50	0.49	*0.69*		
35 C-Self-Discipline	17.28	4.59	50.26	8.45	0.07	−0.49	−0.38	−0.28	−0.37	−0.27	−0.38	−0.49	0.24	0.11	0.08	0.33	0.25	0.19	0.13	0.11	−0.08	−0.01	0.02	0.20	0.28	0.01	−0.02	0.14	−0.04	0.08	0.06	−0.25	0.00	0.85	0.58	0.57	0.68	0.54	*0.78*	
36 C-Deliberation	16.06	4.26	49.85	8.96	0.08	−0.28	−0.13	−0.36	−0.05	0.03	−0.54	−0.25	−0.27	−0.10	−0.28	−0.13	−0.25	−0.21	−0.22	−0.05	−0.18	−0.08	−0.09	−0.18	0.24	0.06	0.18	0.03	0.12	0.18	0.38	0.07	−0.04	0.56	0.29	0.36	0.36	0.13	0.30	*0.72*
37 GPA	2.87	0.60	–	–	−0.33	−0.01	−0.02	−0.03	0.04	−0.03	−0.03	0.03	0.01	0.03	0.08	−0.10	−0.05	0.12	−0.08	−0.20	−0.04	−0.14	−0.10	−0.11	−0.25	−0.09	−0.10	−0.10	−0.15	0.02	−0.06	0.06	−0.20	−0.20	−0.14	−0.01	−0.23	−0.20	−0.22	−0.11

Note. *N* = 230–243. Values printed in italics are internal consistencies (Cronbach’s α). Lower GPA values indicate better performance. |*r*| ≥ 0.13: *p* ≤ 0.05. |*r*| ≥ 0.18: *p* ≤ 0.01. |*r*| ≥ 0.22: *p* ≤ 0.001.

**Table 4:** The overview of the statistically significant effects in Study 2.

Associations of Intelligence and Grade Point Average Were Stronger When Big Five Factors and Facets Were	Region of Significance	Centered Values	
		Min.	Max.
lower on:			
N	<18.37	−43.80	68.46
N2: Angry Hostility	<4.70	−10.93	15.07
N5: Impulsiveness	<4.70	−9.99	11.01
N6: Vulnerability	<3.53	10.78	13.22
O	–	–	–
O4: Actions	<3.27	−8.88	10.12
higher on:			
A	–	–	–
A3: Altruism	>−4.65	−9.71	9.29
A4: Compliance	>−4.85	−13.37	10.63
C	>−15.87	−53.94	49.06
C1: Competence	>−3.74	−10.92	9.08
C3: Dutifulness	>−3.86	−11.71	12.29
C4: Achievement Striving	>−4.27	−8.91	10.09
C5: Self-Discipline	>−4.35	−16.28	10.72
C6: Deliberation	>−4.06	−11.06	9.94

Note. Reading example: the association between intelligence and GPA became statistically significant (*p* < 0.05) when students had higher centered scores on Competence than −3.74. The centered Competence scores ranged from −10.92 to 9.08, with a standard deviation of 3.46 (see Table 3). Thus, the association between intelligence and GPA became insignificant when students scored somewhat more than 1 standard deviation below the Competence mean (*M* = 0).

## Supplementary Materials

Both anonymized data sets in ASCII format as well as the code books are made publicly available at PsychData, the repository of the Leibniz Institute for Psychology Information (ZPID) (doi:10.5160/psychdata.srra08pe02). Both data files can be obtained upon request sent to the ZPID. Test materials cannot be made publicly available due to copyrights.
